# Supplementary material for: Structure of a TRAPPII-Rab11 activation intermediate reveals GTPase substrate selection mechanisms
Source: Sci Adv. 2022 May 13;8(19):eabn7446. doi: 10.1126/sciadv.abn7446 (PMC9106297; doi:10.1126/sciadv.abn7446)
Supplement: Supplementary file 1 — Figs. S1 to S10 Tables S1 to S3 References [file sciadv.abn7446_sm.pdf]

Science Advances
AAAS

Supplementary Materials for

Structure of a TRAPPII-Rab11 activation intermediate reveals GTPase substrate selection mechanisms

Saket R. Bagde and J. Christopher Fromme*

*Corresponding author. Email: jcf14@cornell.edu

Published 13 May 2022, *Sci. Adv.* **8**, eabn7446 (2022)
DOI: 10.1126/sciadv.abn7446

The PDF file includes:

Figs. S1 to S10
Tables S1 to S3
Legend for movie S1
References

Other Supplementary Material for this manuscript includes the following:

Movie S1

A

	TRAPPII	TRAPPIII
Catalytic core subunits	Bet5	Bet5
	Trs20	Trs20
	Bet3	Bet3
	Trs23	Trs23
	Trs31	Trs31
	Trs33	Trs33
	Tca17	-
	Trs120	-
	Trs130	-
	Trs65	-
	-	Trs85

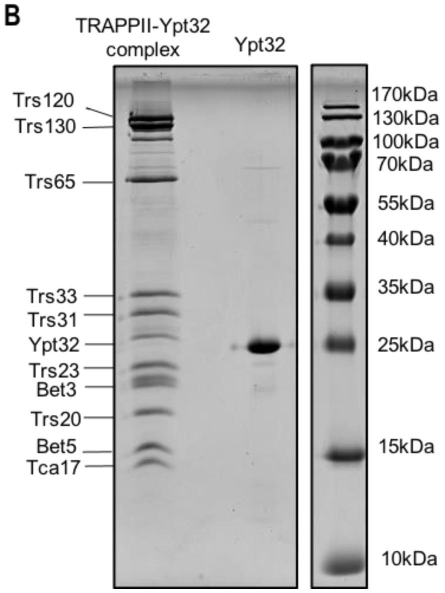

Fig. S1. CryoEM sample preparation.

(**A**) Table detailing the subunit composition of the yeast TRAPPII and TRAPPIII complexes.
(**B**) SDS-PAGE gel showing purified TRAPPII-Rab11/Ypt32 complex and purified Rab11/Ypt32.

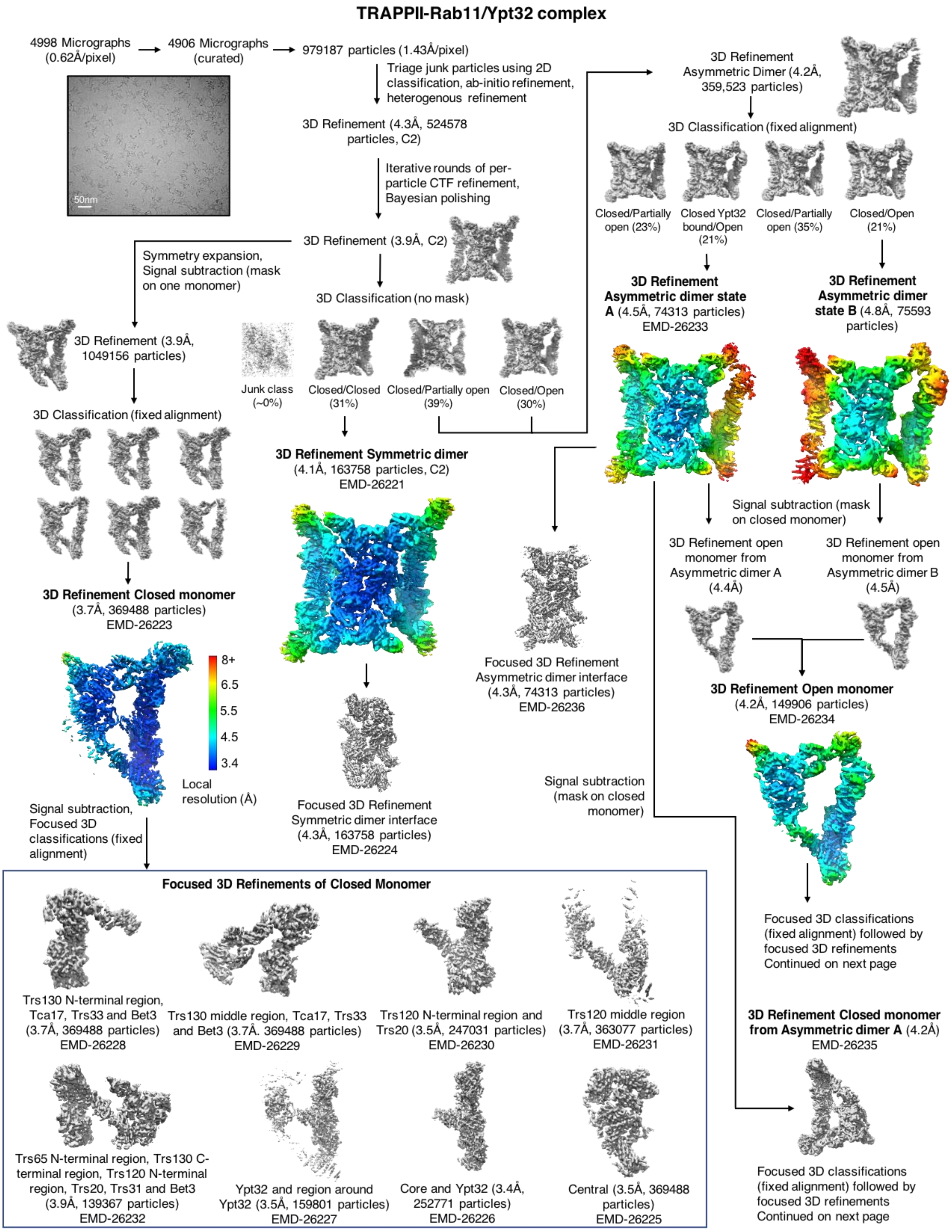
TRAPPII-Rab11/Ypt32 complex
4998 Micrographs (0.62Å/pixel)
4906 Micrographs (curated)
979187 particles (1.43Å/pixel)
50nm
Triage junk particles using 2D classification, ab-initio refinement, heterogenous refinement
3D Refinement (4.3Å, 524578 particles, C2)
Iterative rounds of per-particle CTF refinement, Bayesian polishing
3D Refinement (3.9Å, C2)
Symmetry expansion, Signal subtraction (mask on one monomer)
3D Classification (no mask)
3D Refinement (3.9Å, 1049156 particles)
Junk class (~0%)
Closed/Closed (31%)
Closed/Partially open (39%)
Closed/Open (30%)
3D Classification (fixed alignment)
3D Refinement Symmetric dimer
(4.1Å, 163758 particles, C2)
EMD-26221
3D Refinement Closed monomer
(3.7Å, 369488 particles)
EMD-26223
8+
6.5
5.5
4.5
3.4
Local resolution (Å)
Signal subtraction, Focused 3D classifications (fixed alignment)
Focused 3D Refinement Symmetric dimer interface (4.3Å, 163758 particles) EMD-26224
3D Refinement Asymmetric Dimer (4.2Å, 359,523 particles)
3D Classification (fixed alignment)
Closed/Partially open (23%)
Closed Ypt32 bound/Open (21%)
Closed/Partially open (35%)
Closed/Open (21%)
3D Refinement Asymmetric dimer state A (4.5Å, 74313 particles) EMD-26233
3D Refinement Asymmetric dimer state B (4.8Å, 75593 particles)
Signal subtraction (mask on closed monomer)
3D Refinement open monomer from Asymmetric dimer A (4.4Å)
3D Refinement open monomer from Asymmetric dimer B (4.5Å)
Focused 3D Refinement Asymmetric dimer interface (4.3Å, 74313 particles) EMD-26236
3D Refinement Open monomer
(4.2Å, 149906 particles)
EMD-26234
Signal subtraction (mask on closed monomer)
Focused 3D classifications (fixed alignment) followed by focused 3D refinements Continued on next page
3D Refinement Closed monomer from Asymmetric dimer A (4.2Å)
EMD-26235
Focused 3D classifications (fixed alignment) followed by focused 3D refinements Continued on next page
Focused 3D Refinements of Closed Monomer
Trs130 N-terminal region, Tca17, Trs33 and Bet3 (3.7Å, 369488 particles) EMD-26228
Trs130 middle region, Tca17, Trs33 and Bet3 (3.7Å. 369488 particles) EMD-26229
Trs120 N-terminal region and Trs20 (3.5Å, 247031 particles) EMD-26230
Trs120 middle region (3.7Å, 363077 particles) EMD-26231
Trs65 N-terminal region, Trs130 C-terminal region, Trs120 N-terminal region, Trs20, Trs31 and Bet3 (3.9Å, 139367 particles) EMD-26232
Ypt32 and region around Ypt32 (3.5Å, 159801 particles) EMD-26227
Core and Ypt32 (3.4Å, 252771 particles) EMD-26226
Central (3.5Å, 369488 particles) EMD-26225

Continued from previous page

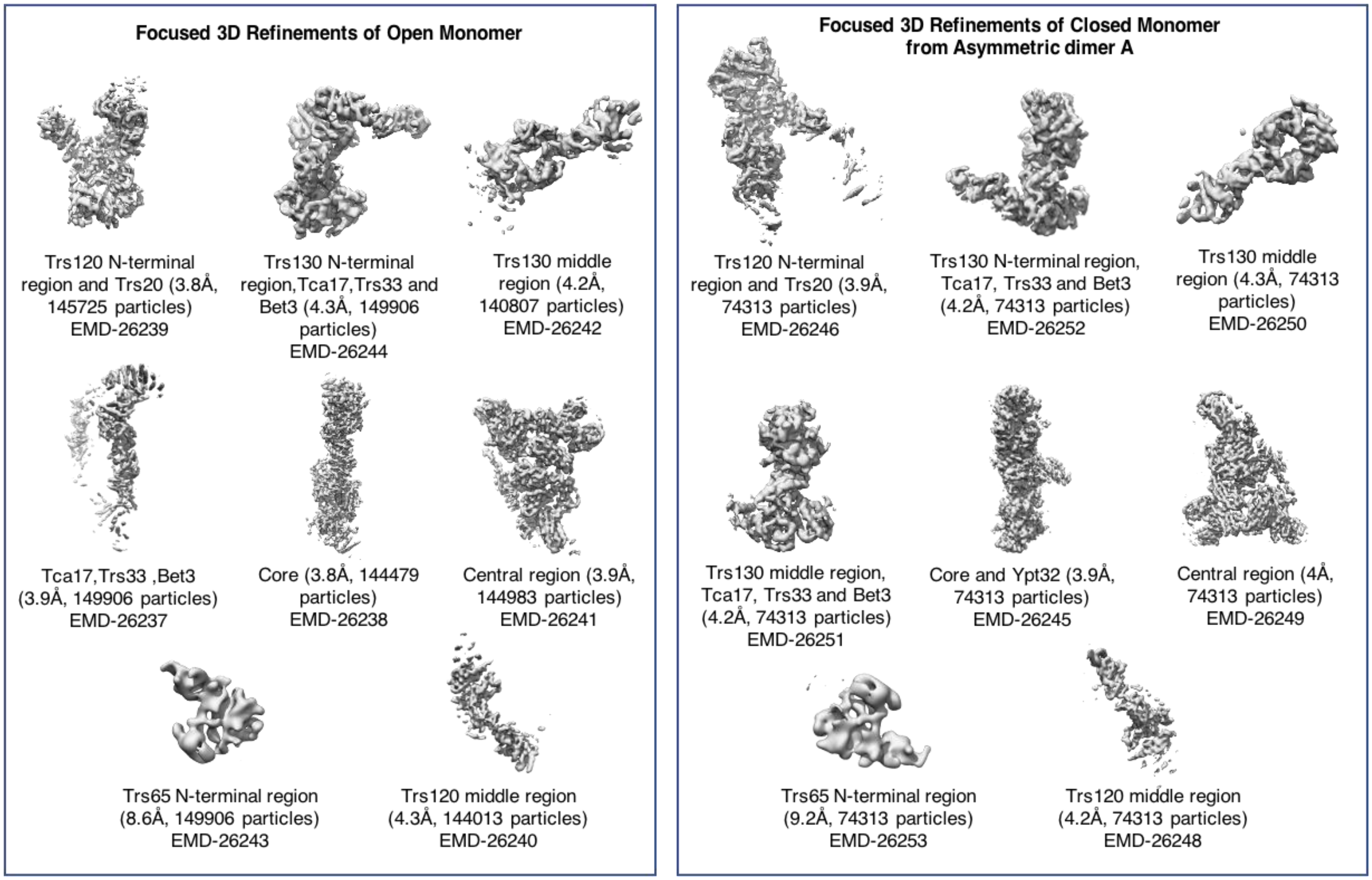

Fig. S2. CryoEM data processing pipeline for TRAPPII-Rab11/Ypt32 complex.

Flowchart illustrating the data processing strategy for the TRAPPII-Rab11/Ypt32 complex cryoEM data (see Methods).

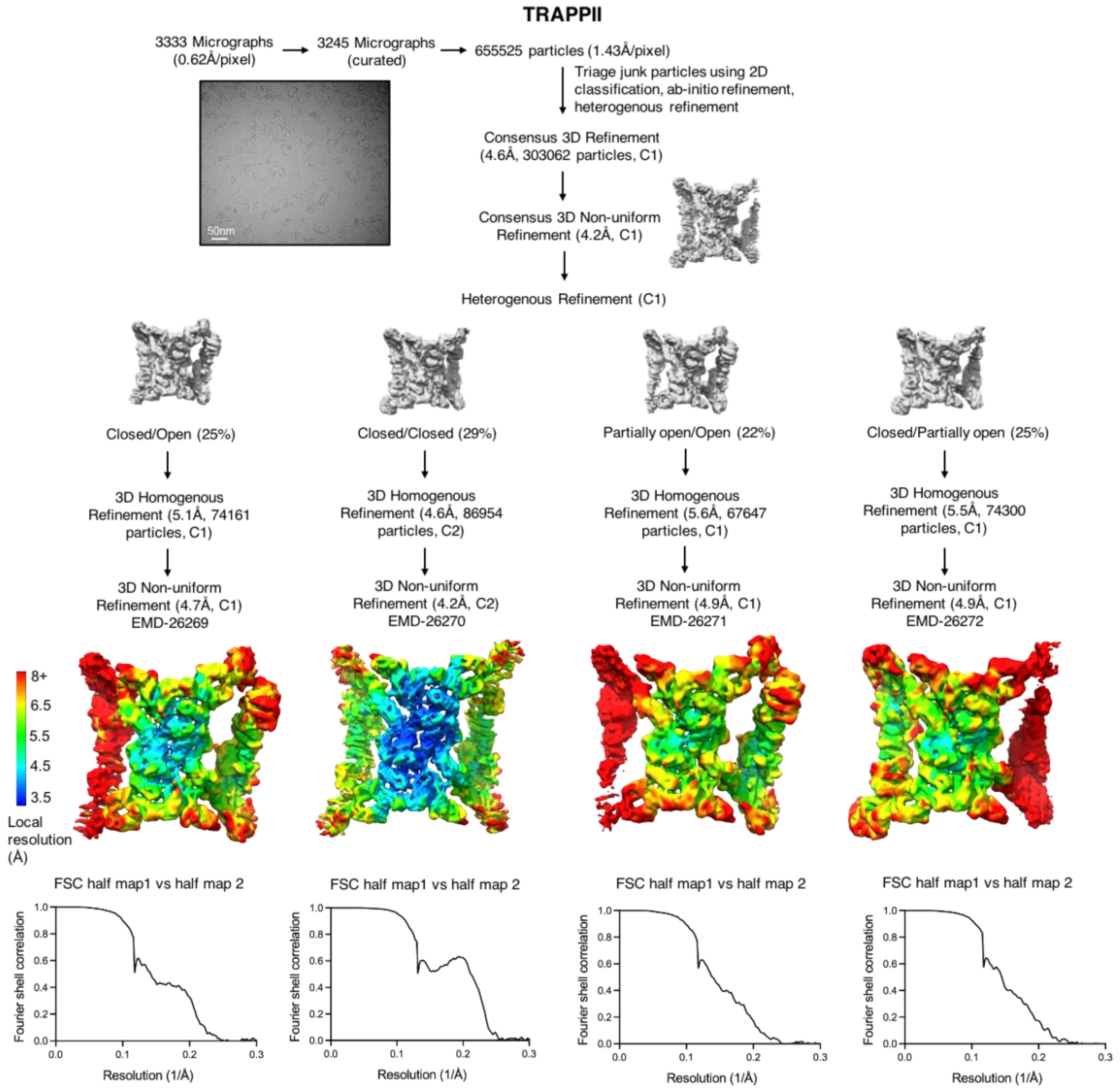

Fig. S3. CryoEM data processing pipeline for TRAPPII.

Flowchart illustrating the data processing strategy for the TRAPPII-only cryoEM data (see Methods). Fourier shell correlation plots for the indicated refinement reconstructions are shown below the maps.

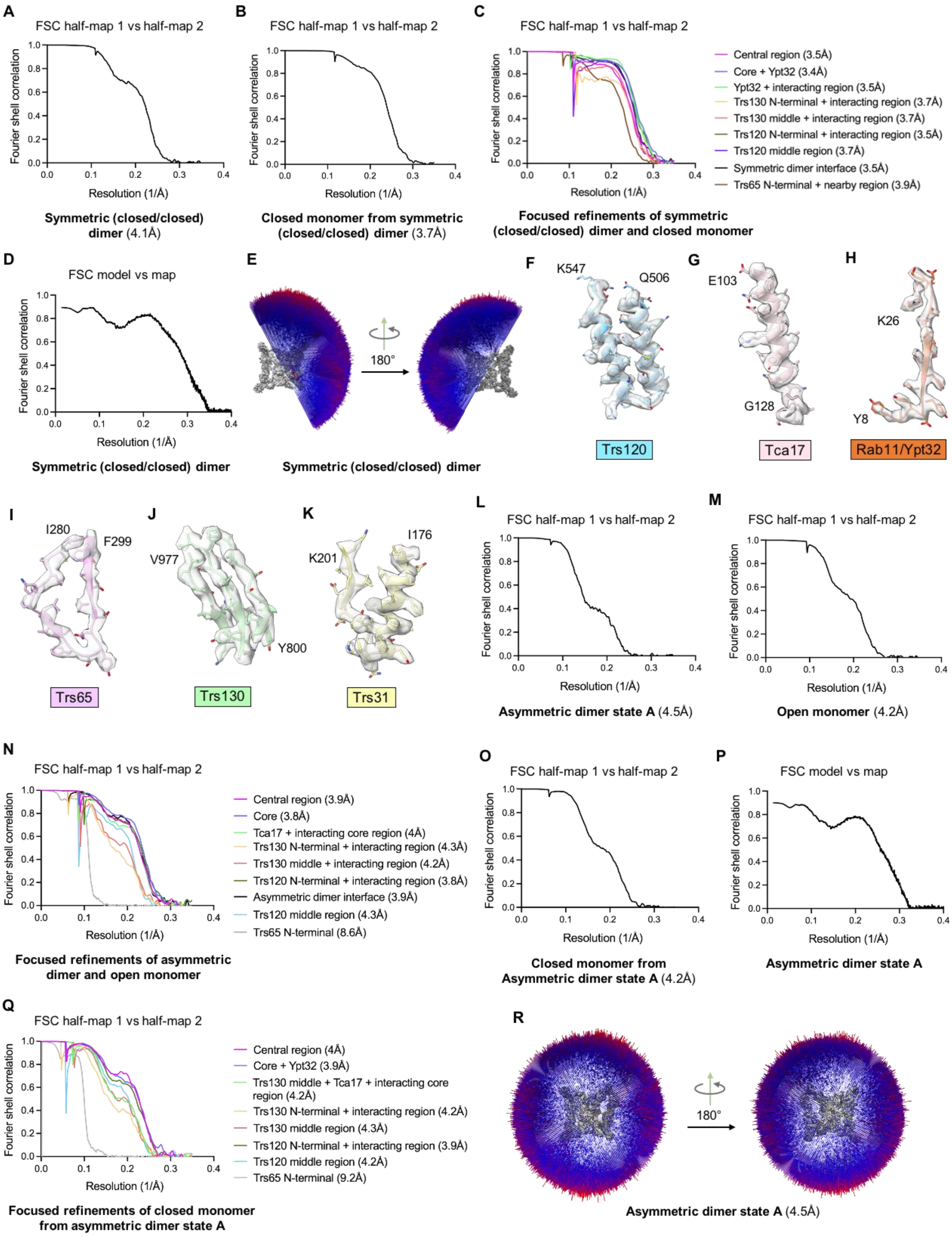
A
FSC half-map 1 vs half-map 2
Fourier shell correlation
Resolution (1/Å)
Symmetric (closed/closed) dimer (4.1Å)
B
FSC half-map 1 vs half-map 2
Fourier shell correlation
Resolution (1/Å)
Closed monomer from symmetric (closed/closed) dimer (3.7Å)
C
FSC half-map 1 vs half-map 2
Fourier shell correlation
Resolution (1/Å)
Central region (3.5Å)
Core + Ypt32 (3.4Å)
Ypt32 + interacting region (3.5Å)
Trs130 N-terminal + interacting region (3.7Å)
Trs130 middle + interacting region (3.7Å)
Trs120 N-terminal + interacting region (3.5Å)
Trs120 middle region (3.7Å)
Symmetric dimer interface (3.5Å)
Trs65 N-terminal + nearby region (3.9Å)
Focused refinements of symmetric (closed/closed) dimer and closed monomer
D
FSC model vs map
Fourier shell correlation
Resolution (1/Å)
Symmetric (closed/closed) dimer
E
180°
Symmetric (closed/closed) dimer
F
K547
Q506
Trs120
G
E103
G128
Tca17
H
K26
Y8
Rab11/Ypt32
I
I280
F299
Trs65
J
V977
Y800
Trs130
K
K201
I176
Trs31
L
FSC half-map 1 vs half-map 2
Fourier shell correlation
Resolution (1/Å)
Asymmetric dimer state A (4.5Å)
M
FSC half-map 1 vs half-map 2
Fourier shell correlation
Resolution (1/Å)
Open monomer (4.2Å)
N
FSC half-map 1 vs half-map 2
Fourier shell correlation
Resolution (1/Å)
Central region (3.9Å)
Core (3.8Å)
Tca17 + interacting core region (4Å)
Trs130 N-terminal + interacting region (4.3Å)
Trs130 middle + interacting region (4.2Å)
Trs120 N-terminal + interacting region (3.8Å)
Asymmetric dimer interface (3.9Å)
Trs120 middle region (4.3Å)
Trs65 N-terminal (8.6Å)
Focused refinements of asymmetric dimer and open monomer
O
FSC half-map 1 vs half-map 2
Fourier shell correlation
Resolution (1/Å)
Closed monomer from Asymmetric dimer state A (4.2Å)
P
FSC model vs map
Fourier shell correlation
Resolution (1/Å)
Asymmetric dimer state A
Q
FSC half-map 1 vs half-map 2
Fourier shell correlation
Resolution (1/Å)
Central region (4Å)
Core + Ypt32 (3.9Å)
Trs130 middle + Tca17 + interacting core region (4.2Å)
Trs130 N-terminal + interacting region (4.2Å)
Trs130 middle region (4.3Å)
Trs120 N-terminal + interacting region (3.9Å)
Trs120 middle region (4.2Å)
Trs65 N-terminal (9.2Å)
Focused refinements of closed monomer from asymmetric dimer state A
R
180°
Asymmetric dimer state A (4.5Å)

Fig. S4. CryoEM map quality and model-map fit.

(**A**) Fourier shell correlation plot for the symmetric (closed/closed) TRAPPII-Rab11/Ypt32 dimer reconstruction. (**B**) Fourier shell correlation plot for the symmetry-expanded TRAPPII-Rab11/Ypt32 closed monomer reconstruction. (**C**) Fourier shell correlation plots for the indicated focused refinement reconstructions produced from the symmetric (closed/closed) TRAPPII-Rab11/Ypt32 complex. (**D**) Fourier shell correlation plot comparing the refined symmetric (closed/closed) TRAPPII-Rab11/Ypt32 dimer model to the symmetric (closed/closed) TRAPPII-Rab11/Ypt32 dimer reconstruction. (**E**) Orientation histogram for the symmetric (closed/closed) TRAPPII-Rab11/Ypt32 dimer particle refinement. (**F**) Example cryoEM density for the Trs120 subunit. (**G**) Example cryoEM density for the Tca17 subunit. (**H**) Example cryoEM density for Rab11/Ypt32. (**I**) Example cryoEM density for the Trs65 subunit. (**J**) Example cryoEM density for the Trs130 subunit. (**K**) Example cryoEM density for the Trs31 subunit (as an example of the core complex). (**L**) Fourier shell correlation plot for the asymmetric (closed/open) dimer "state A" reconstruction. (**M**) Fourier shell correlation plot for the open TRAPPII monomer reconstruction. (**N**) Fourier shell correlation plots for the indicated focused refinement reconstructions produced from the open monomer. (**O**) Fourier shell correlation plot for the closed TRAPPII monomer reconstruction from the asymmetric (closed/open) dimer "state A". (**P**) Fourier shell correlation plot comparing the refined symmetric asymmetric dimer model to the asymmetric dimer reconstruction. (**Q**) Fourier shell correlation plots for the indicated focused refinement reconstructions produced from the closed monomer of the asymmetric (closed/open) dimer "state A". (**R**) Orientation histogram for the asymmetric dimer "state A" particle refinement.

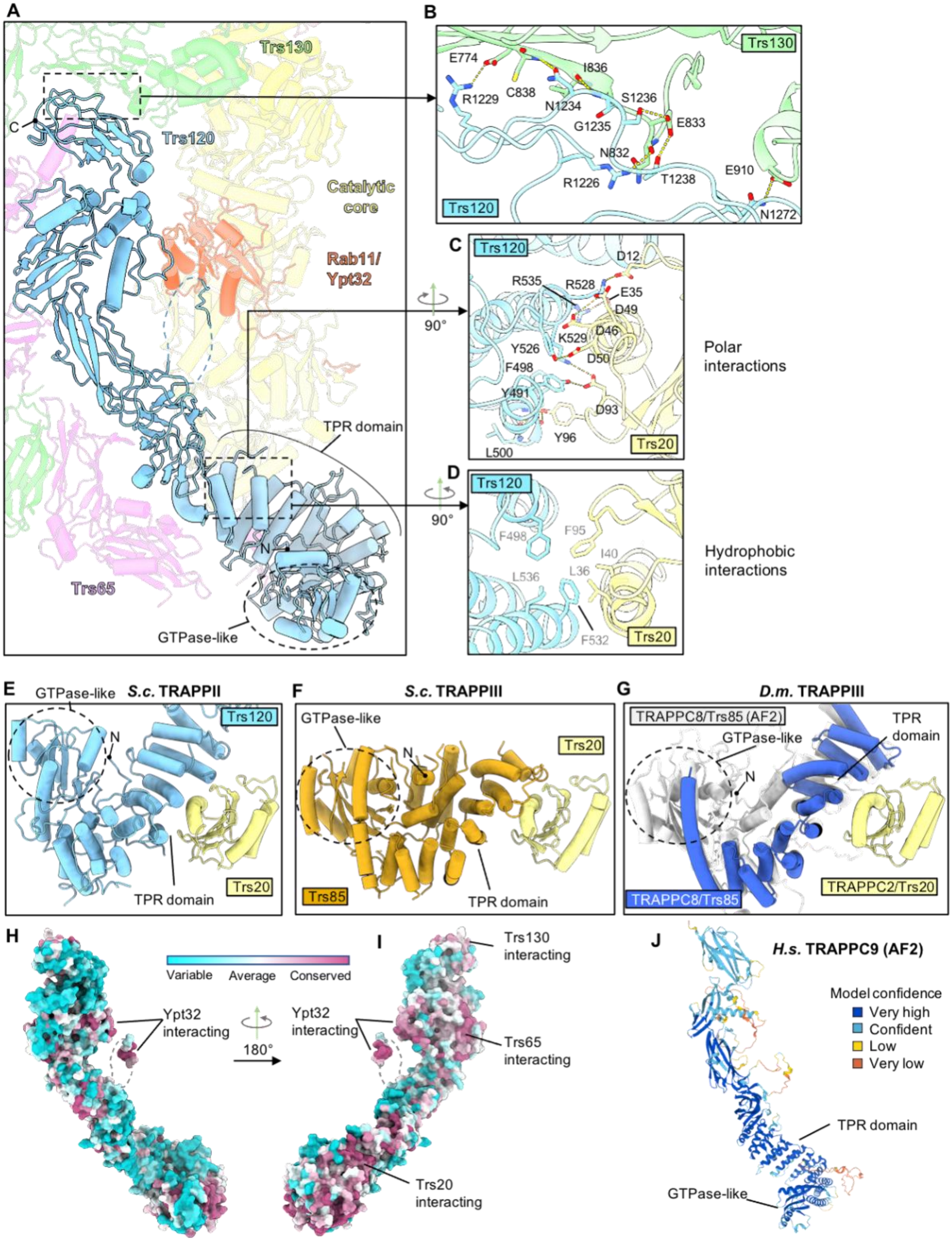

Fig. S5. Analysis of Trs120 structure and interactions within the TRAPPII complex.

(**A**) Overall fold of Trs120. TPR : tetratricopeptide repeat. (**B**) Interactions at the Trs120-Trs130 interface. (**C**) Polar interactions at the Trs120-Trs20 interface. (**D**) Hydrophobic interactions at the

Trs120-Trs20 interface. (**E-G**) Comparison of Trs120-Trs20 in TRAPPII (E, this work), Trs85-Trs20 in yeast TRAPPIII (*25*) (F), and TRAPPC8-TRAPPC2 in fly TRAPPIII (*26*) (G) interfaces. Note that the GTPase-like domain of TRAPPC8 was not modeled in the published fly TRAPPIII structure, so we superimposed the AlphaFold prediction (*53*) (shown in gray) onto the fly TRAPPC8 experimental model (*26*). (**H,I**) Conservation analysis of Trs120. (**J**) AlphaFold prediction of the human TRAPPC9 structure (*53*) shows an overall fold similar to that of Trs120.

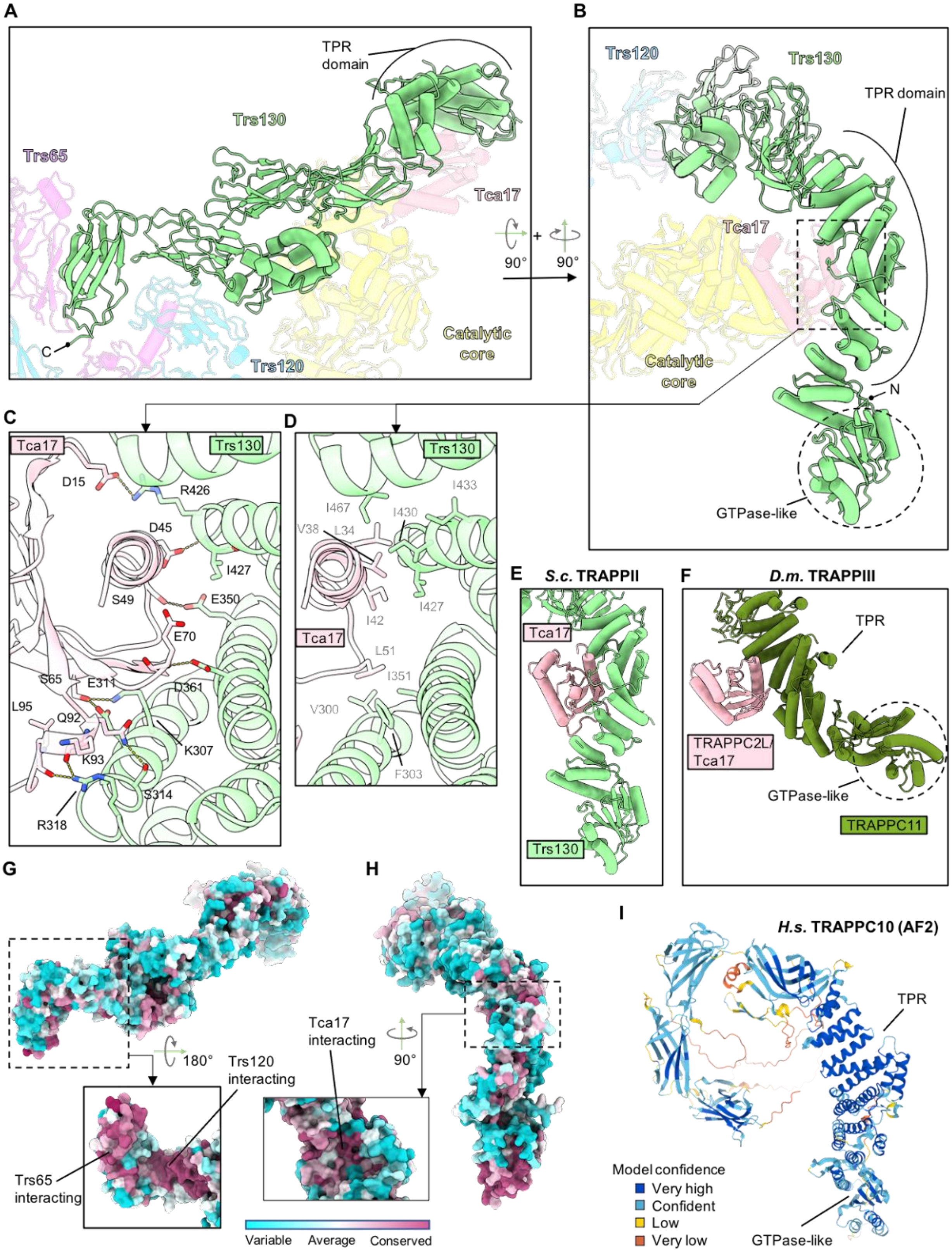

A
TPR domain
Trs130
Trs65
Tca17
C
Trs120
Catalytic core
90°
90°
B
Trs120
Trs130
TPR domain
Tca17
Catalytic core
N
GTPase-like
C
Tca17
Trs130
D15
R426
D45
I427
S49
E350
E70
S65
E311
D361
L95
Q92
K93
K307
S314
R318
D
Trs130
I433
I467
I430
V38
L34
I427
I42
Tca17
L51
I351
V300
F303
E
S.c. TRAPPII
Tca17
Trs130
F
D.m. TRAPPIII
TPR
TRAPPC2L/Tca17
GTPase-like
TRAPPC11
G
180°
Trs120 interacting
Trs65 interacting
H
Tca17 interacting
90°
Variable
Average
Conserved
I
H.s. TRAPPC10 (AF2)
TPR
Model confidence
Very high
Confident
Low
Very low
GTPase-like

Fig. S6. Analysis of Trs130 structure and interactions within the TRAPPII complex.

(**A**,**B**) Overall fold of Trs130. TPR : tetratricopeptide repeat. (**C**) Polar interactions at the Trs130-Tca17 interface. Tca17 residue D45 is equivalent to a mutation in human TRAPPC2L associated with a neurodevelopmental disorder (*36*) (**D**) Hydrophobic interactions at the Trs130-Tca17 interface. (**E**,**F**) Comparison of the leg elements from Trs130 (E, this work) and TRAPPC11 from fly TRAPPIII (*26*) (F). Note that the leg of TRAPPC11 from fly TRAPPIII appears "bent" relative to the "straight" leg of Trs130. (**G**,**H**) Conservation analysis of Trs130. (**I**) AlphaFold structure prediction (*53*) of the human TRAPPII subunit TRAPPC10 (Trs130 paralog) shows an overall fold similar to that of Trs130. Note that the TRAPPC10 leg appears "straight", similar to the structure of yeast Trs130 but unlike the corresponding region of TRAPPC11 which appears "bent" in fly TRAPPIII.

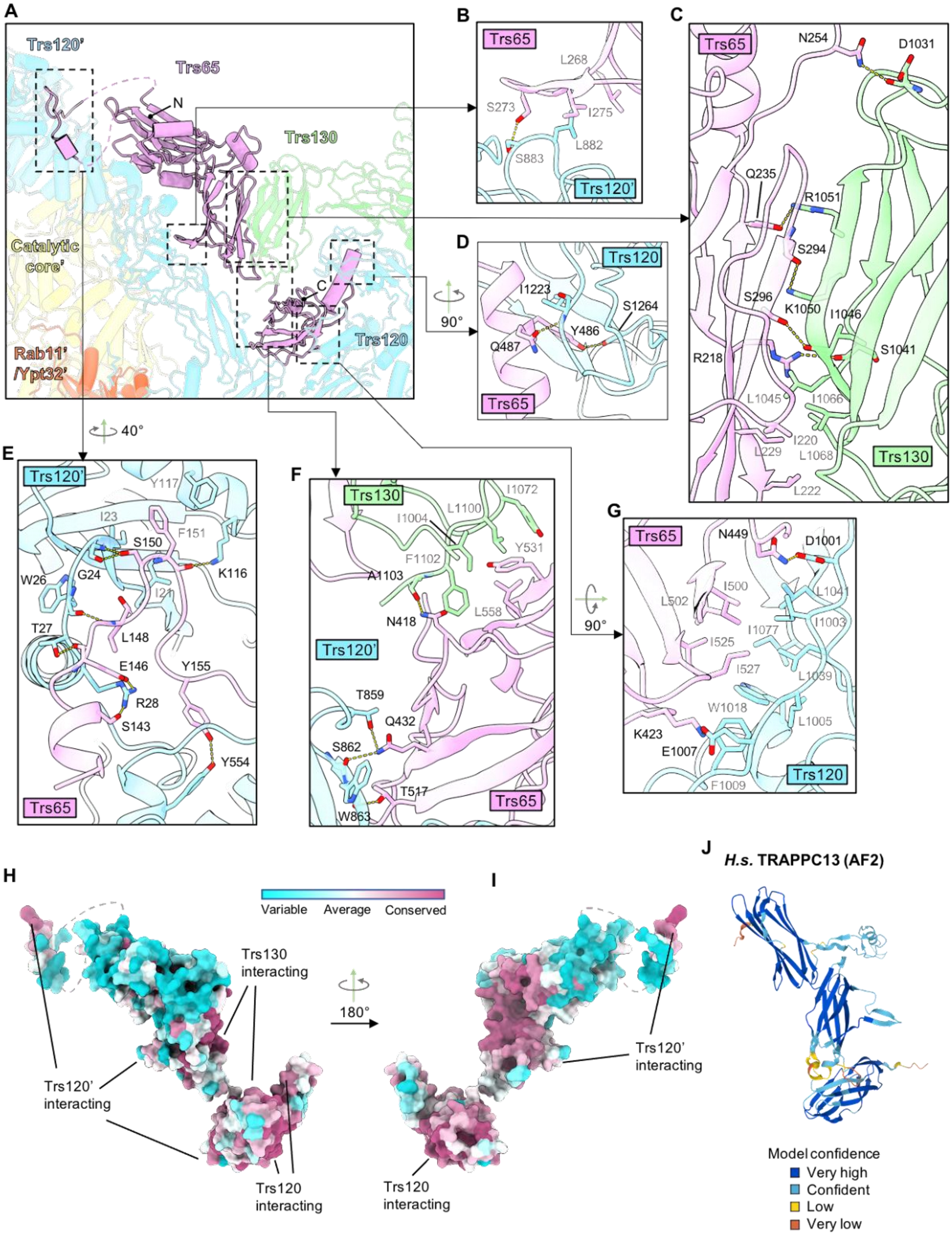

A
Trs120'
Trs65
Trs130
Catalytic core'
Rab11' /Ypt32'
Trs120
N
C
40°
90°
B
Trs65
L268
S273
I275
L882
S883
Trs120'
C
Trs65
N254
D1031
Q235
R1051
S294
S296
K1050
I1046
S1041
R218
L1045
I1066
I220
L229
L1068
L222
Trs130
D
Trs120
I1223
S1264
Y486
Q487
Trs65
E
Trs120'
Y117
I23
S150
F151
K116
W26
G24
I21
T27
L148
E146
Y155
R28
S143
Y554
Trs65
F
Trs130
I1072
L1100
I1004
Y531
F1102
A1103
L558
N418
Trs120'
T859
Q432
S862
T517
Trs65
W863
G
Trs65
N449
D1001
L502
I500
L1041
I1003
I1077
I525
I527
L1039
W1018
L1005
K423
E1007
Trs120
F1009
H
Variable
Average
Conserved
Trs130 interacting
Trs120' interacting
Trs120 interacting
180°
I
Trs120' interacting
Trs120 interacting
J
H.s. TRAPPC13 (AF2)
Model confidence
Very high
Confident
Low
Very low

Fig. S7. Analysis of Trs65 structure and interactions within the TRAPPII complex.

(**A**) Overall fold of Trs65. Labels with prime (‘) symbols indicate subunits belonging to the other monomer within the TRAPPII dimer. (**B-G**) Interactions between Trs65 and other subunits. (**H,I**) Conservation analysis of Trs65. (**J**) AlphaFold structure prediction (*53*) of human TRAPPC13 shows an overall fold similar to that of Trs65. Note that although it exhibits structural similarity to Trs65, TRAPPC13 is part of the metazoan TRAPPIII complex (*30*, *40*).

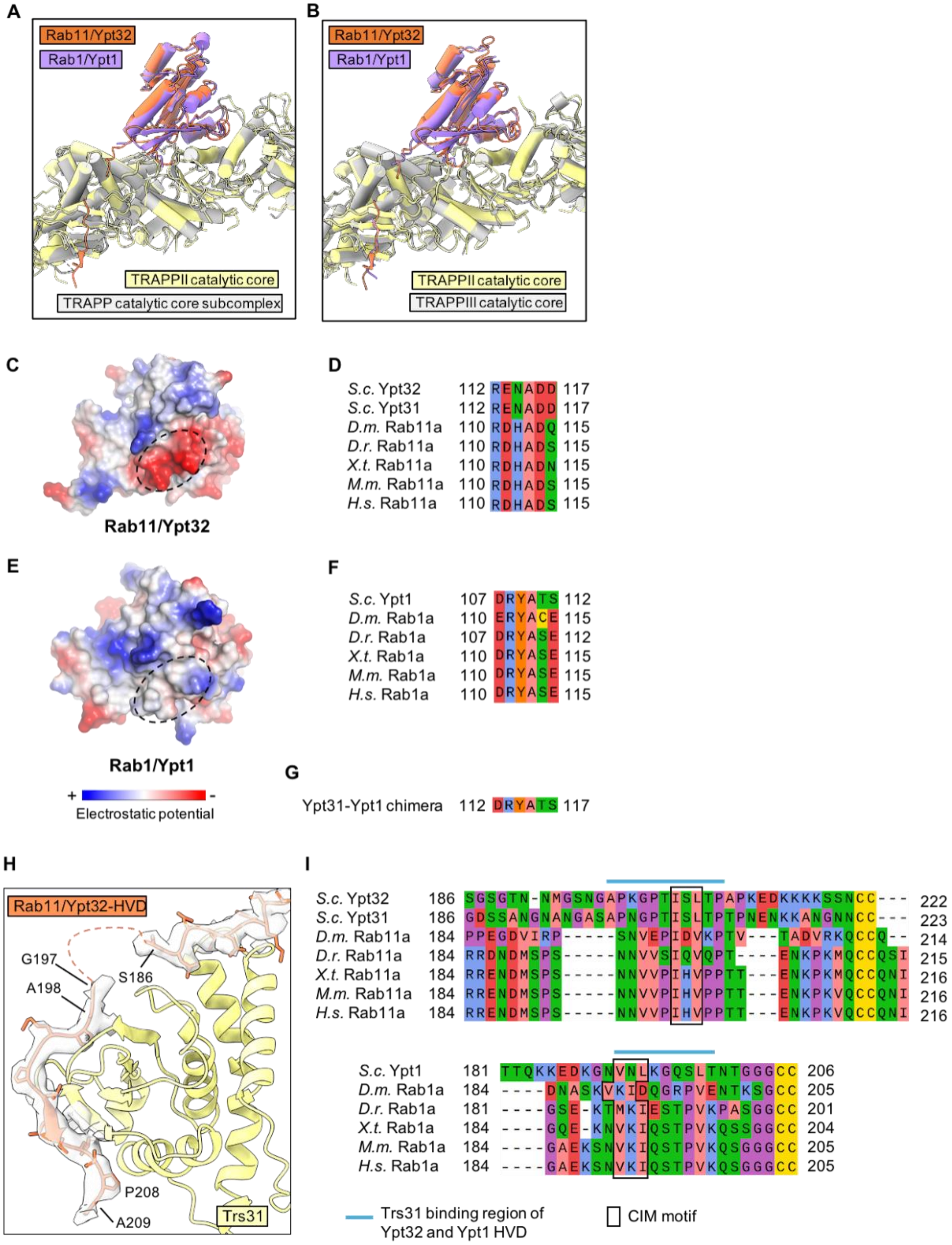
A
Rab11/Ypt32
Rab1/Ypt1
TRAPPII catalytic core
TRAPP catalytic core subcomplex
B
Rab11/Ypt32
Rab1/Ypt1
TRAPPII catalytic core
TRAPPIII catalytic core
C
Rab11/Ypt32
D
S.c. Ypt32 112 RENADD 117
S.c. Ypt31 112 RENADD 117
D.m. Rab11a 110 RDHADQ 115
D.r. Rab11a 110 RDHADS 115
X.t. Rab11a 110 RDHADN 115
M.m. Rab11a 110 RDHADS 115
H.s. Rab11a 110 RDHADS 115
E
Rab1/Ypt1
+ -
Electrostatic potential
F
S.c. Ypt1 107 DRYATS 112
D.m. Rab1a 110 ERYACE 115
D.r. Rab1a 107 DRYASE 112
X.t. Rab1a 110 DRYASE 115
M.m. Rab1a 110 DRYASE 115
H.s. Rab1a 110 DRYASE 115
G
Ypt31-Ypt1 chimera 112 DRYATS 117
H
Rab11/Ypt32-HVD
G197
A198
S186
P208
A209
Trs31
I
S.c. Ypt32 186 SGSGTN-NMGSNGAPKGPTISLTPAPKEDKKKKSSNCC--- 222
S.c. Ypt31 186 GDSSANGNANGASAPNGPTISLTPTPNENKKANGNNCC--- 223
D.m. Rab11a 184 PPEGDVIRP-----SNVEPIDVKPTV---TADVRKQCCQ-- 214
D.r. Rab11a 184 RRDNDMSPS-----NNVVSIQVQPT----ENKPKMQCCQSI 215
X.t. Rab11a 184 RRENDMSPS-----NNVVPIHVPPTT---ENKPKMQCCQNI 216
M.m. Rab11a 184 RRENDMSPS-----NNVVPIHVPPTT---ENKPKVQCCQNI 216
H.s. Rab11a 184 RRENDMSPS-----NNVVPIHVPPTT---ENKPKVQCCQNI 216
S.c. Ypt1 181 TTQKKEDKGNVNLKGQSLTNTGGGCC 206
D.m. Rab1a 184 ----DNASKVKIDQGRPVENTKSGCC 205
D.r. Rab1a 181 ----GSE-KTMKIESTPVKPASGGCC 201
X.t. Rab1a 184 ----GQE-KNVKIQSTPVKQSSGGCC 204
M.m. Rab1a 184 ----GAEKSNVKIQSTPVKQSGGGCC 205
H.s. Rab1a 184 ----GAEKSNVKIQSTPVKQSGGGCC 205
Trs31 binding region of Ypt32 and Ypt1 HVD
CIM motif

Fig. S8. Differences between Rab1 and Rab11 TRAPP core binding interactions.

(**A**) Superposition comparing the structure of TRAPPII-bound Rab11/Ypt32 (this study) to TRAPP core-bound Rab1/Ypt1 (*22*). (**B**) Superposition comparing the structure of TRAPPII-bound Rab11/Ypt32 (this study) to TRAPPIII bound Rab1/Ypt1 (*25*). (**C-F**) Comparison of the surfaces of Rab11/Ypt32 and Rab1/Ypt1 to highlight the electrostatic differences (**C,E**) of sequences (**D,F**) located near the TRAPP-core binding site of each Rab. (**G**) Sequence of the region used in the Ypt31-Ypt1 graft chimera construct used for experiments shown in Fig. 2, F and G. (**H**) Close-up of the interaction between the Rab11/Ypt32 HVD (orange) and the Trs31 core subunit in TRAPPII (yellow). CryoEM density of the HVD is shown in white with black outline. (**I**) Alignments of the Rab1 and Rab11 HVD regions are aligned to each other using the Rab1/Ypt1 and Rab11/Ypt32 binding-sites on Trs31 (blue line) as a structural alignment reference. The CIM motifs required for prenylation are indicated.

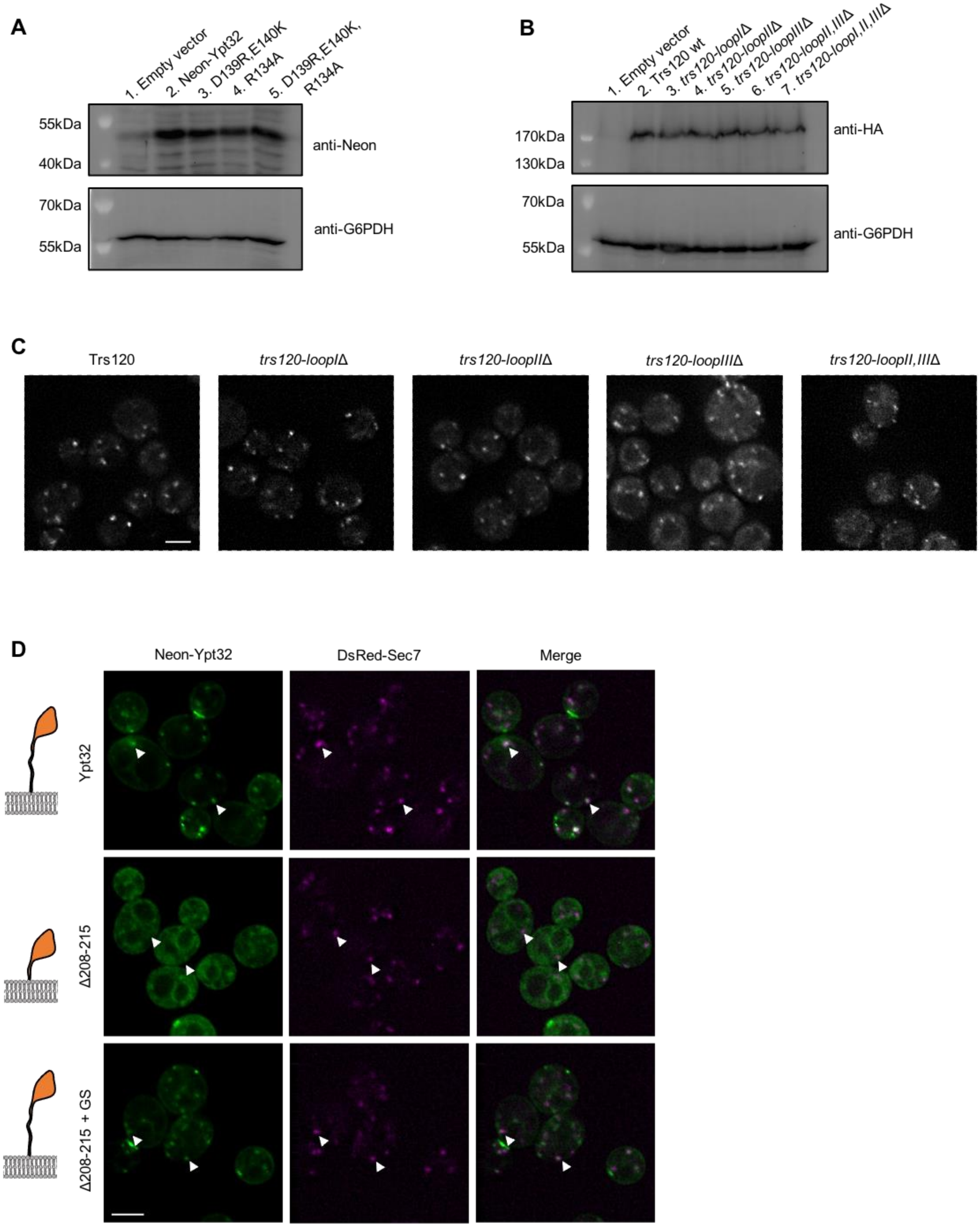
A
1. Empty vector
2. Neon-Ypt32
3. D139R,E140K
4. R134A
5. D139R,E140K, R134A
55kDa
40kDa
anti-Neon
70kDa
55kDa
anti-G6PDH
B
1. Empty vector
2. Trs120 wt
3. trs120-loopIΔ
4. trs120-loopIIΔ
5. trs120-loopIIIΔ
6. trs120-loopII,IIIΔ
7. trs120-loopI,II,IIIΔ
170kDa
130kDa
anti-HA
70kDa
55kDa
anti-G6PDH
C
Trs120
trs120-loopIΔ
trs120-loopIIΔ
trs120-loopIIIΔ
trs120-loopII,IIIΔ
D
Neon-Ypt32
DsRed-Sec7
Merge
Ypt32
Δ208-215
Δ208-215 + GS

Fig. S9. Western blot analysis of Rab11/Ypt32 mutants and localization of Trs120 and Rab11/Ypt32 mutants.

(**A**) Immunoblot to confirm expression of the mutants shown in Fig. 3E. (**B**) Immunoblot to confirm expression of the HA-tagged Trs120 and loop mutants shown in Fig. 3F. (**C**) Imaging data using Trs120-mNeonGreen constructs indicating that the Trs120 loop deletion constructs are expressed and localized similar to the wild-type. (**D**) Localization of an extra copy of wild-type and mutant mNeonGreen-Rab11/Ypt32 constructs in yeast. Colocalization with Sec7-marked late-Golgi compartments is indicative of active Rab11/Ypt32. Scale bars shown in (C) and (D) are 2μm.

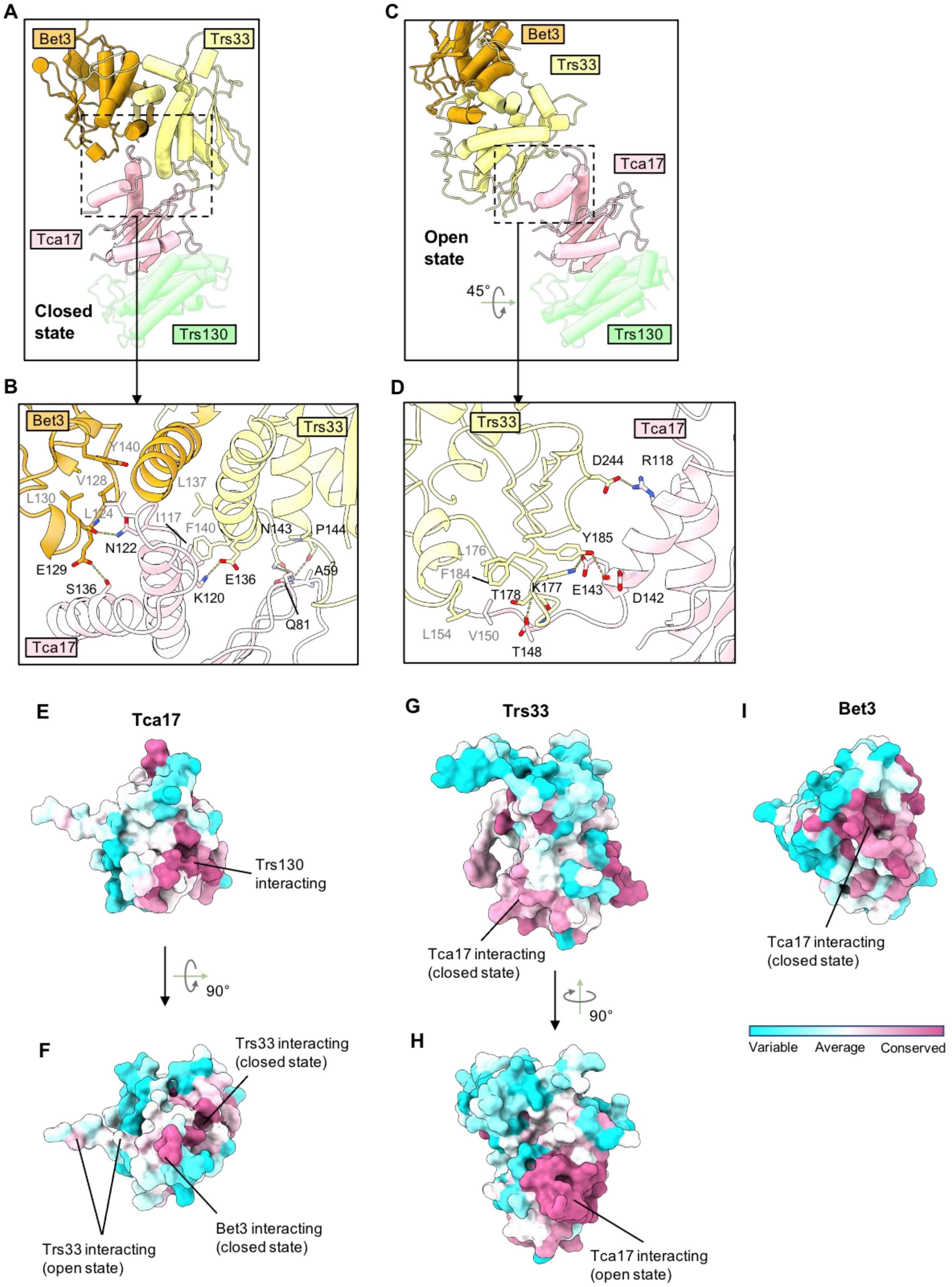

A
Bet3
Trs33
Tca17
Closed state
Trs130
B
Bet3
Trs33
Y140
V128
L137
L130
L124
I117
F140
N143
P144
N122
E129
E136
A59
S136
K120
Q81
Tca17
C
Bet3
Trs33
Tca17
Open state
45°
Trs130
D
Trs33
Tca17
D244
R118
Y185
L176
F184
K177
E143
T178
D142
L154
V150
T148
E
Tca17
Trs130 interacting
90°
F
Trs33 interacting (closed state)
Trs33 interacting (open state)
Bet3 interacting (closed state)
G
Trs33
Tca17 interacting (closed state)
90°
H
Tca17 interacting (open state)
I
Bet3
Tca17 interacting (closed state)
Variable
Average
Conserved

Fig. S10. Distinct interactions between Tca17 and the core in the open and closed states.

(**A,B**) Close-up view of the interactions between Tca17 and the core subunits Trs33 and Bet3 in the closed state. (**C,D**) Close-up view of the interactions between Tca17 and Trs33 in the open state. Note that Trs33 F184 corresponds to the residue position of a substitution mutation in human TRAPPC6A that is associated with a neurodevelopmental syndrome (*39*) (the disease allele results in a TRAPPC6A Y93N substitution according to the UNIPROT database sequence of the human protein). (**E,F**) Conservation analysis of Tca17, indicating the interaction sites. (**G,H**) Conservation analysis of Trs33. (**I**) Conservation analysis of Bet3.

Table S1. CryoEM data collection and model validation statistics for TRAPPII-Rab11/Ypt32 complex.

	Closed/closed state			Closed/open state			
	**Symmetric dimer (composite structure) (EMD-26254) (PDB 7U05)**	**Symmetric dimer (consensus map) (EMD-26221)**	**Closed monomer (EMD-26223)**	**Asymmetric dimer state A (composite structure) (EMD-26255) (PDB 7U06)**	**Asymmetric dimer state A (consensus map) (EMD-26233)**	**Open monomer (EMD-26234)**	**Closed monomer from asymmetric dimer state A (EMD-26235)**
**Data collection and processing**							
**Magnification**	63000						
**Voltage (kV)**	200						
**Electron exposure (e–/Å^2)**	53.2						
**Defocus range (μm)**	-0.8 to -2.5						
**Pixel size (Å)**	1.24						
**Total number of movies**	4998						
**Symmetry imposed**	C2	C2	C1	C1	C1	C1	C1
**Initial particle images (no.)**	524,578	524,578	524,578	524,578	524,578	524,578	524,578
**Final particle images (no.)**	NA	163,758	369,488	NA	74,313	149,906	74,313
**Map resolution (Å) FSC threshold (0.143)**	NA	4.1	3.7	NA	4.5	4.2	4.2
**Map resolution range (Å)**	NA	3.7 - 12.9	3.5 - 9.7	NA	4 - 15.1	4 - 10.7	4 - 11.5
**Refinement**							
**Initial model used (PDB code)**	3CUE, 3RWO, 3PR6			3CUE, 3RWO, 3PR6			
**Model resolution (Å) FSC threshold (0.5)**	3.5			3.8			

**Map sharpening *B* factor (Å^2)**	NA	-94	-81	NA	-92	-77	-87
**Model composition**<br>**Non-hydrogen atoms**<br>**Protein residues**<br>**Ligands**	 65,598<br>8,350<br>PLM: 4			 63,860<br>8,151<br>PLM: 4			
***B* factors (Å^2)**<br>**Protein**<br>**Ligand**	 104.00<br>92.49			 109.33<br>92.93			
**R.m.s. deviations**<br>**Bond lengths (Å)**<br>**Bond angles (°)**	 0.004<br>0.818			 0.004<br>0.824			
**Validation**<br>**MolProbity score**<br>**Clashscore**<br>**Poor rotamers (%)**	 1.74<br>4.22<br>0.06			 1.80<br>4.95<br>0.03			
**Ramachandran plot**<br>**Favored (%)**<br>**Allowed (%)**<br>**Disallowed (%)**	 90.61<br>9.28<br>0.11			 90.28<br>9.57<br>0.15			

Table S2. CryoEM data collection and map statistics for TRAPPII-only complex.

	Closed/open state (consensus map) (EMD-26269)	Closed/closed state (consensus map) (EMD-26270)	Partially open/open state (consensus map) (EMD-26271)	Closed/Partially open (consensus map) (EMD-26272)
**Data collection and processing**				
**Magnification**	63000			
**Voltage (kV)**	200			
**Electron exposure (e–/Å^2)**	54			
**Defocus range (μm)**	-0.8 to -2.5			
**Pixel size (Å)**	1.24			
**Total number of movies**	3333			
**Symmetry imposed**	C1	C2	C1	C1
**Initial particle images (no.)**	303,062	303,062	303,062	303,062
**Final particle images (no.)**	74,161	86,954	67,647	74,300
**Map resolution (Å) FSC threshold (0.143)**	4.7	4.2	4.9	4.9
**Map resolution range (Å)**	3.1 - 30	3.1 - 12	3.1 - 15.7	3.1 - 13.9
**Map sharpening B factor (Å^2)**	-120.1	-108.7	-153.7	-154

Table S3. Strains and plasmids used in this study.

Yeast strains	Reference/Source
CFY1904: MATa his3-D1 leu2-D0 met15-D0 ura3-D0 Trs130-TAP::HIS3	Dharmacon (cat# YSC1178-202232849)
CFY2449: SEY6210.1 Trs85-mNeonGreen::HIS3	(19)
CFY1638: SEY6210.1 ypt31D::KanMX ypt32D::KanMX::NatMX + VSB283	(13)
CFY1992: SEY6210.5 trs120Δ::HIS3/TRS120	(13)
CFY2148: SEY6210.1 trs120Δ::HIS3 + pCF1285	This study
CFY2451: SEY6210.1 Trs130-mNeonGreen::HIS3	(21)
CFY1993: SEY6210.5 trs130Δ::HIS3/TRS130	(13)
CFY2150: SEY6210.1 trs130Δ::HIS3 + pCF1286	This study
CFY4633: SEY6210.1 trs130Δ::HIS3 + pSB12	This study
CFY4634: SEY6210.1 trs130Δ::HIS3 + pSB13	This study
CFY1681: SEY6210.1 Sec7-6xDsRed::URA3	(7)
**Plasmids**	**Source**
pGEX-6P-GST-Ypt32-His$_7$	A. Bretscher (Cornell University, Ithaca, NY)
pLT77: pRS416-mRFPmars-Ypt1(D124N)-Fis1	(23)
pLT75: pRS415-mRFPmars-Ypt31(D129N)-Fis1	(23)
pSB1 (Ypt31-Ypt1 chimera): pRS415-mRFPmars-Ypt31($_{112}$RENADD>DRYATS)-Fis1	This paper
pLT116: pRS415-mNeonGreen-Ypt32	(23)
pSB2: pRS415-mNeonGreen-Ypt32($_{139}$DE>RK)	This paper
pSB2: pRS415-mNeonGreen-Ypt32(R134A)	This paper
pSB2: pRS415-mNeonGreen-Ypt32(R134A; $_{139}$DE>RK)	This paper
pSB2: pRS415-Trs120-mNeonGreen-3XHA	This paper
pSB2 (Trs120(loopIΔ)): pRS415-Trs120(Δ696-704+GS)-mNeonGreen-3XHA	This paper
pSB3 (Trs120(loopIIΔ)): pRS415-Trs120(Δ901-910+GSGSG)-mNeonGreen-3XHA	This paper
pSB4 (Trs120(loopIIIΔ)): pRS415-Trs120(Δ1093-1110+GSGS)-mNeonGreen-3XHA	This paper
pSB5 (Trs120(loopII,IIIΔ)): pRS415-Trs120(Δ901-910+GSGSG; Δ1093-1110+GSGS)-mNeonGreen-3XHA	This paper
pSB6 (Trs120(loopIΔI,II,IIIΔ)): pRS415-Trs120(Δ696-704+GS; Δ901-910+GSGSG; Δ1093-1110+GSGS)-mNeonGreen-3XHA	This paper
pLT85: pRS415-mRFPmars-Ypt32(D129N)-Fis1	(23)
pSB7: pRS415-mRFPmars-Ypt32(D129N; $_{139}$DE>RK)-Fis1	This paper
pSB8: pRS415-mNeonGreen-Ypt32(Δ208-215)	This paper

pSB9: pRS415-mNeonGreen-Ypt32(Δ208-215+GSGSSGSG)	This paper
pSB10: pRS415-mRFPmars-Ypt32(D129N; Δ208-215)-Fis1	This paper
pSB11: pRS415-mRFPmars-Ypt32(D129N; Δ208-215+GSGSSGSG)-Fis1	This paper
pSB12: pRS415-Trs130-mNeonGreen-3XHA	This paper
pSB13: pRS415-Trs130(Δ201-340)-mNeonGreen-3XHA	This paper
VSB283: pRS416-Ypt31	(13)
pCF1286: pRS416-Trs130	This paper
pCF1285: pRS416-Trs120	This paper
pRS415	(54)
pRS416	(54)

Movie S1. Mechanism of activation of Rab11/Ypt32 by the TRAPPII complex.

Coloring is the same as in Fig. 1. At the beginning of the movie, a TRAPPII monomer in the closed conformation is viewed from the side. The movie then shows a "morph" transition to the open conformation. In the next stage, GDP-bound Rab11/Ypt32 diffuses into the active site chamber and binds to the catalytic core. The structure then transitions back to the closed conformation, triggering conformational change of Rab11/Ypt32 and GDP release. GTP then binds to Rab11/Ypt32, as the concentration of GTP is significantly higher than the concentration of GDP within cells. GTP-binding triggers another conformational change of Rab11/Ypt32 to its active state. This conformation of Rab11/Ypt32 is incompatible with stable binding to the core. Transition to the open state of TRAPPII enables activated GTP-bound Rab11/Ypt32 to diffuse away from the active site chamber, and this TRAPPII monomer is available for another round of nucleotide exchange. This movie was made using Chimera. Note that morphing transitions are for illustrative purposes and do not necessarily represent the actual conformation transition pathways.

REFERENCES AND NOTES

1. A.-C. Borchers, L. Langemeyer, C. Ungermann, Who's in control? Principles of Rab GTPase activation in endolysosomal membrane trafficking and beyond. *J. Cell Biol.* **220**, e202105120 (2021).

2. S. R. Pfeffer, Rab GTPases: Master regulators that establish the secretory and endocytic pathways. *Mol. Biol. Cell* **28**, 712–715 (2017).

3. N. Morozova, Y. Liang, A. A. Tokarev, S. H. Chen, R. Cox, J. Andrejic, Z. Lipatova, V. A. Sciorra, S. D. Emr, N. Segev, TRAPPII subunits are required for the specificity switch of a Ypt-Rab GEF. *Nat. Cell Biol.* **8**, 1263–1269 (2006).

4. X. Cao, N. Ballew, C. Barlowe, Initial docking of ER-derived vesicles requires Uso1p and Ypt1p but is independent of SNARE proteins. *EMBO J.* **17**, 2156–2165 (1998).

5. H. Plutner, A. D. Cox, S. Pind, R. Khosravi-Far, J. R. Bourne, R. Schwaninger, C. J. Der, W. E. Balch, Rab1b regulates vesicular transport between the endoplasmic reticulum and successive Golgi compartments. *J. Cell Biol.* **115**, 31–43 (1991).

6. N. Segev, Mediation of the attachment or fusion step in vesicular transport by the GTP-binding Ypt1 protein. *Science* **252**, 1553–1556 (1991).

7. C. M. McDonold, J. C. Fromme, Four GTPases differentially regulate the Sec7 Arf-GEF to direct traffic at the trans-golgi network. *Dev. Cell* **30**, 759–767 (2014).

8. M. Benli, F. Döring, D. G. Robinson, X. Yang, D. Gallwitz, Two GTPase isoforms, Ypt31p and Ypt32p, are essential for Golgi function in yeast. *EMBO J.* **15**, 6460–6475 (1996).

9. G. Jedd, J. Mulholland, N. Segev, Two new Ypt GTPases are required for exit from the yeast trans-Golgi compartment. *J. Cell Biol.* **137**, 563–580 (1997).

10. T. Welz, J. Wellbourne-Wood, E. Kerkhoff, Orchestration of cell surface proteins by Rab11. *Trends Cell Biol.* **24**, 407–415 (2014).

11. A. Sakaguchi, M. Sato, K. Sato, K. Gengyo-Ando, T. Yorimitsu, J. Nakai, T. Hara, K. Sato, K. Sato, REI-1 is a guanine nucleotide exchange factor regulating RAB-11 localization and function in *C. elegans* embryos. *Dev. Cell* **35**, 211–221 (2015).

12. B. Xiong, V. Bayat, M. Jaiswal, K. Zhang, H. Sandoval, W.-L. Charng, T. Li, G. David, L. Duraine, Y.-Q. Lin, G. G. Neely, S. Yamamoto, H. J. Bellen, Crag is a GEF for Rab11 required for rhodopsin trafficking and maintenance of adult photoreceptor cells. *PLoS Biol.* **10**, e1001438 (2012).

13. V. A. Sciorra, A. Audhya, A. B. Parsons, N. Segev, C. Boone, S. D. Emr, Synthetic genetic array analysis of the PtdIns 4-kinase Pik1p identifies components in a Golgi-specific Ypt31/rab-GTPase signaling pathway. *Mol. Biol. Cell* **16**, 776–793 (2005).

14. C. C. Robinett, M. G. Giansanti, M. Gatti, M. T. Fuller, TRAPPII is required for cleavage furrow ingression and localization of Rab11 in dividing male meiotic cells of Drosophila. *J. Cell Sci.* **122**, 4526–4534 (2009).

15. Y. Otsuka, T. Satoh, N. Nakayama, R. Inaba, H. Yamashita, A. K. Satoh, Parcas is the predominant Rab11-GEF for rhodopsin transport in *Drosophila* photoreceptors. *J. Cell Sci.* **132**, jcs231431 (2019).

16. C. M. Klinger, M. J. Klute, J. B. Dacks, Comparative genomic analysis of multi-subunit tethering complexes demonstrates an ancient pan-eukaryotic complement and sculpting in Apicomplexa. *PLOS ONE* **8**, e76278 (2013).

17. F. Riedel, A. Galindo, N. Muschalik, S. Munro, The two TRAPP complexes of metazoans have distinct roles and act on different Rab GTPases. *J. Cell Biol.* **217**, 601–617 (2018).

18. M. A. Lynch-Day, D. Bhandari, S. Menon, J. Huang, H. Cai, C. R. Bartholomew, J. H. Brumell, S. Ferro-Novick, D. J. Klionsky, Trs85 directs a Ypt1 GEF, TRAPPIII, to the phagophore to promote autophagy. *Proc. Natl. Acad. Sci. U.S.A.* **107**, 7811–7816 (2010).

19. L. L. Thomas, A. M. N. Joiner, J. C. Fromme, The TRAPPIII complex activates the GTPase Ypt1 (Rab1) in the secretory pathway. *J. Cell Biol.* **217**, 283–298 (2018).

20. M. Sacher, N. Shahrzad, H. Kamel, M. P. Milev, TRAPPopathies: An emerging set of disorders linked to variations in the genes encoding transport protein particle (TRAPP)-associated proteins. *Traffic* **20**, 5–26 (2019).

21. L. L. Thomas, J. C. Fromme, GTPase cross talk regulates TRAPPII activation of Rab11 homologues during vesicle biogenesis. *J. Cell Biol.* **215**, 499–513 (2016).

22. Y. Cai, H. F. Chin, D. Lazarova, S. Menon, C. Fu, H. Cai, A. Sclafani, D. W. Rodgers, E. M. De La Cruz, S. Ferro-Novick, K. M. Reinisch, The structural basis for activation of the Rab Ypt1p by the TRAPP membrane-tethering complexes. *Cell* **133**, 1202–1213 (2008).

23. L. L. Thomas, S. A. van der Vegt, J. C. Fromme, A steric gating mechanism dictates the substrate specificity of a Rab-GEF. *Dev. Cell* **48**, 100–114.e9 (2019).

24. Y.-G. Kim, S. Raunser, C. Munger, J. Wagner, Y.-L. Song, M. Cygler, T. Walz, B.-H. Oh, M. Sacher, The architecture of the multisubunit TRAPP I complex suggests a model for vesicle tethering. *Cell* **127**, 817–830 (2006).

25. A. M. Joiner, B. P. Phillips, K. Yugandhar, E. J. Sanford, M. B. Smolka, H. Yu, E. A. Miller, J. C. Fromme, Structural basis of TRAPPIII-mediated Rab1 activation. *EMBO J.* **40**, e107607 (2021).

26. A. Galindo, V. J. Planelles-Herrero, G. Degliesposti, S. Munro, Cryo-EM structure of metazoan TRAPPIII, the multi-subunit complex that activates the GTPase Rab1. *EMBO J.* **40**, e107608 (2021).

27. M. L. Jenkins, N. J. Harris, U. Dalwadi, K. D. Fleming, D. S. Ziemianowicz, A. Rafiei, E. M. Martin, D. C. Schriemer, C. K. Yip, J. E. Burke, The substrate specificity of the human TRAPPII complex's Rab-guanine nucleotide exchange factor activity. *Commun Biol.* **3**, 735 (2020).

28. C. K. Yip, J. Berscheminski, T. Walz, Molecular architecture of the TRAPPII complex and implications for vesicle tethering. *Nat. Struct. Mol. Biol.* **17**, 1298–1304 (2010).

29. M. Pinar, E. Arias-Palomo, V. de Los Ríos, H. N. Arst Jr, M. A. Peñalva, Characterization of *Aspergillus nidulans* TRAPPs uncovers unprecedented similarities between fungi and metazoans and reveals the modular assembly of TRAPPII. *PLOS Genet.* **15**, e1008557 (2019).

30. C. Choi, M. Davey, C. Schluter, P. Pandher, Y. Fang, L. J. Foster, E. Conibear, Organization and assembly of the TRAPPII complex. *Traffic* **12**, 715–725 (2011).

31. B. Montpetit, E. Conibear, Identification of the novel TRAPP associated protein Tca17. *Traffic* **10**, 713–723 (2009).

32. M. Zong, X.-G. Wu, C. W. L. Chan, M. Y. Choi, H. C. Chan, J. A. Tanner, S. Yu, The adaptor function of TRAPPC2 in mammalian TRAPPs explains TRAPPC2-associated SEDT and TRAPPC9-associated congenital intellectual disability. *PLOS ONE* **6**, e23350 (2011).

33. D. Taussig, Z. Lipatova, J. J. Kim, X. Zhang, N. Segev, Trs20 is required for TRAPP II assembly. *Traffic* **14**, 678–690 (2013).

34. S. Brunet, N. Shahrzad, D. Saint-Dic, H. Dutczak, M. Sacher, A trs20 mutation that mimics an SEDT-causing mutation blocks selective and non-selective autophagy: A model for TRAPP III organization. *Traffic* **14**, 1091–1104 (2013).

35. I. R. Humphreys, J. Pei, M. Baek, A. Krishnakumar, I. Anishchenko, S. Ovchinnikov, J. Zhang, T. J. Ness, S. Banjade, S. R. Bagde, V. G. Stancheva, X.-H. Li, K. Liu, Z. Zheng, D. J. Barrero, U. Roy, J. Kuper, I. S. Fernández, B. Szakal, D. Branzei, J. Rizo, C. Kisker, E. C. Greene, S. Biggins, S. Keeney, E. A. Miller, J. C. Fromme, T. L. Hendrickson, Q. Cong, D. Baker, Computed structures of core eukaryotic protein complexes. *Science* **374**, eabm4805 (2021).

36. M. P. Milev, C. Graziano, D. Karall, W. F. E. Kuper, N. Al-Deri, D. M. Cordelli, T. B. Haack, K. Danhauser, A. Iuso, F. Palombo, T. Pippucci, H. Prokisch, D. Saint-Dic, M. Seri, D. Stanga, G. Cenacchi, K. L. I. van Gassen, J. Zschocke, C. Fauth, J. A. Mayr, M. Sacher, P. M. van Hasselt, Bi-allelic mutations in *TRAPPC2L* result in a neurodevelopmental disorder and have an impact on RAB11 in fibroblasts. *J. Med. Genet.* **55**, 753–764 (2018).

37. A. Sultana, Y. Jin, C. Dregger, E. Franklin, L. S. Weisman, A. R. Khan, The activation cycle of Rab GTPase Ypt32 reveals structural determinants of effector recruitment and GDI binding. *FEBS Lett.* **585**, 3520–3527 (2011).

38. A. Rak, O. Pylypenko, A. Niculae, K. Pyatkov, R. S. Goody, K. Alexandrov, Structure of the Rab7:REP-1 complex: Insights into the mechanism of Rab prenylation and choroideremia disease. *Cell* **117**, 749–760 (2004).

39. H. S. Mohamoud, S. Ahmed, M. Jelani, N. Alrayes, K. Childs, N. Vadgama, M. M. Almramhi, J. Y. Al-Aama, S. Goodbourn, J. Nasir, A missense mutation in TRAPPC6A leads to build-up of the protein, in patients with a neurodevelopmental syndrome and dysmorphic features. *Sci. Rep.* **8**, 2053 (2018).

40. M. C. Bassik, M. Kampmann, R. J. Lebbink, S. Wang, M. Y. Hein, I. Poser, J. Weibezahn, M. A. Horlbeck, S. Chen, M. Mann, A. A. Hyman, E. M. Leproust, M. T. McManus, J. S. Weissman, A systematic mammalian genetic interaction map reveals pathways underlying ricin susceptibility. *Cell* **152**, 909–922 (2013).

41. S. Q. Zheng, E. Palovcak, J.-P. Armache, K. A. Verba, Y. Cheng, D. A. Agard, MotionCor2: Anisotropic correction of beam-induced motion for improved cryo-electron microscopy. *Nat. Methods* **14**, 331–332 (2017).

42. A. Punjani, J. L. Rubinstein, D. J. Fleet, M. A. Brubaker, cryoSPARC: Algorithms for rapid unsupervised cryo-EM structure determination. *Nat. Methods* **14**, 290–296 (2017).

43. J. Zivanov, T. Nakane, B. O. Forsberg, D. Kimanius, W. J. Hagen, E. Lindahl, S. H. Scheres, New tools for automated high-resolution cryo-EM structure determination in RELION-3. *eLife* **7**, e42166 (2018).

44. J. Zivanov, T. Nakane, S. H. W. Scheres, Estimation of high-order aberrations and anisotropic magnification from cryo-EM data sets in -3.1. *IUCrJ.* **7**, 253–267 (2020).

45. T. C. Terwilliger, S. J. Ludtke, R. J. Read, P. D. Adams, P. V. Afonine, Improvement of cryo-EM maps by density modification. *Nat. Methods* **17**, 923–927 (2020).

46. D. Liebschner, P. V. Afonine, M. L. Baker, G. Bunkóczi, V. B. Chen, T. I. Croll, B. Hintze, L. W. Hung, S. Jain, A. J. McCoy, N. W. Moriarty, R. D. Oeffner, B. K. Poon, M. G. Prisant, R. J. Read, J. S. Richardson, D. C. Richardson, M. D. Sammito, O. V. Sobolev, D. H. Stockwell, T. C. Terwilliger, A. G. Urzhumtsev, L. L. Videau, C. J. Williams, P. D. Adams, Macromolecular structure determination using

x-rays, neutrons and electrons: Recent developments in Phenix. *Acta Crystallogr D Struct Biol.* **75**, 861–877 (2019).

47. E. F. Pettersen, T. D. Goddard, C. C. Huang, G. S. Couch, D. M. Greenblatt, E. C. Meng, T. E. Ferrin, UCSF Chimera—A visualization system for exploratory research and analysis. *J. Comput. Chem.* **25**, 1605–1612 (2004).

48. P. Emsley, B. Lohkamp, W. G. Scott, K. Cowtan, Features and development of Coot. *Acta Crystallogr. D Biol. Crystallogr.* **66**, 486–501 (2010).

49. J. Yang, I. Anishchenko, H. Park, Z. Peng, S. Ovchinnikov, D. Baker, Improved protein structure prediction using predicted interresidue orientations. *Proc. Natl. Acad. Sci. U.S.A.* **117**, 1496–1503 (2020).

50. P. V. Afonine, B. P. Klaholz, N. W. Moriarty, B. K. Poon, O. V. Sobolev, T. C. Terwilliger, P. D. Adams, A. Urzhumtsev, New tools for the analysis and validation of cryo-EM maps and atomic models. *Acta Crystallogr. D Struct. Biol.* **74**, 814–840 (2018).

51. A. Morin, B. Eisenbraun, J. Key, P. C. Sanschagrin, M. A. Timony, M. Ottaviano, P. Sliz, Collaboration gets the most out of software. *eLife* **2**, e01456 (2013).

52. H. Ashkenazy, S. Abadi, E. Martz, O. Chay, I. Mayrose, T. Pupko, N. Ben-Tal, ConSurf 2016: An improved methodology to estimate and visualize evolutionary conservation in macromolecules. *Nucleic Acids Res.* **44**, W344–W350 (2016).

53. J. Jumper, R. Evans, A. Pritzel, T. Green, M. Figurnov, O. Ronneberger, K. Tunyasuvunakool, R. Bates, A. Žídek, A. Potapenko, A. Bridgland, C. Meyer, S. A. A. Kohl, A. J. Ballard, A. Cowie, B. Romera-Paredes, S. Nikolov, R. Jain, J. Adler, T. Back, S. Petersen, D. Reiman, E. Clancy, M. Zielinski, M. Steinegger, M. Pacholska, T. Berghammer, S. Bodenstein, D. Silver, O. Vinyals, A. W. Senior, K. Kavukcuoglu, P. Kohli, D. Hassabis, Highly accurate protein structure prediction with AlphaFold. *Nature* **596**, 583–589 (2021).

54. R. S. Sikorski, P. Hieter, A system of shuttle vectors and yeast host strains designed for efficient manipulation of DNA in *Saccharomyces cerevisiae*. *Genetics* **122**, 19–27 (1989).
